# BLAMM: BLAS-based algorithm for finding position weight matrix occurrences in DNA sequences on CPUs and GPUs

**DOI:** 10.1186/s12859-020-3348-6

**Published:** 2020-03-11

**Authors:** Jan Fostier

**Affiliations:** Department of Information Technology - IDLab, Ghent University - imec, Technologiepark 126, Ghent (Zwijnaarde), B-9052 Belgium

**Keywords:** Position weight matrix (PWM), High performance computing (HPC), Basic linear algebra subprograms (BLAS), Graphics processing units (GPUs)

## Abstract

**Background:**

The identification of all matches of a large set of position weight matrices (PWMs) in long DNA sequences requires significant computational resources for which a number of efficient yet complex algorithms have been proposed.

**Results:**

We propose BLAMM, a simple and efficient tool inspired by high performance computing techniques. The workload is expressed in terms of matrix-matrix products that are evaluated with high efficiency using optimized BLAS library implementations. The algorithm is easy to parallelize and implement on CPUs and GPUs and has a runtime that is independent of the selected *p*-value. In terms of single-core performance, it is competitive with state-of-the-art software for PWM matching while being much more efficient when using multithreading. Additionally, BLAMM requires negligible memory. For example, both strands of the entire human genome can be scanned for 1404 PWMs in the JASPAR database in 13 min with a *p*-value of 10^−4^ using a 36-core machine. On a dual GPU system, the same task can be performed in under 5 min.

**Conclusions:**

BLAMM is an efficient tool for identifying PWM matches in large DNA sequences. Its C++ source code is available under the GNU General Public License Version 3 at https://github.com/biointec/blamm.

## Background

Position weight matrices (PWM), also referred to as a position-specific scoring matrices (PSSM), are used to model short, biologically relevant sequence patterns such as transcription factor binding sites [1]. PWMs offer more flexibility than consensus patterns as they can allow variation at each position in the pattern. Databases such as JASPAR [2], UniPROBE [3] and TRANSFAC [4] host large collections of curated PWMs.

Position weight matrices are generated from an alignment of functionally related sequences. In this work we focus on DNA sequences. Assuming independence between positions, these alignments can be summarized in a 4×*m* position frequency matrix (PFM), with *m* the length of the alignment, where each matrix element PFM (*i*,*j*) represents the frequency of character *i* (0=‘A’; 1=‘C’; 2=‘G’; 3=‘T’) at position *j* in the alignment. By computing, at each position, the relative occurrence of each nucleotide, the position probability matrix (PPM) is derived:

1 $$ \text{PPM}(i,j) = \frac{\text{PFM}(i,j)+\alpha}{\sum_{i}(\text{PFM}(i,j)+\alpha)}  $$

where *α*≥0 is a pseudocount that acts as a smoothing parameter to avoid zero probabilities [5]. Finally, when computing the logarithm (base-2) of the ratio of the PPM elements and the corresponding background nucleotide probability *b*_*i*_, the PWM is obtained:

2 $$ \text{PWM}(i,j) = \log_{2}\left(\frac{\text{PPM}(i,j)}{b_{i}}\right)  $$

**Fig. 1:**
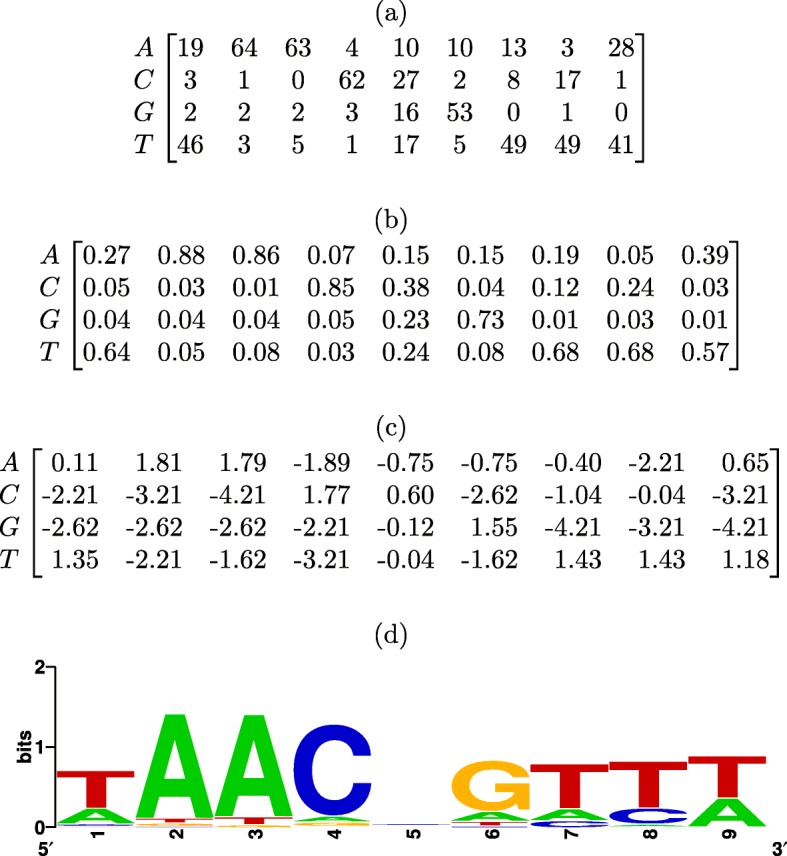
Position frequency matrix (PFM), position probability matrix (PPM), position weight matrix (PWM) and sequence logo of the MYB.Ph3 transcription factor. The PFM was obtained from the JASPAR database [2]. The PPM/PWM were computed using a pseudocount *α*=1 and assuming a uniform background nucleotide composition (*b*_*i*_=0.25). The sequence logo was generated using WebLogo [32]. **a** Position Frequency Matrix (PFM). **b** Position Probability Matrix (PPM). **c** Position Weight Matrix (PWM). **d** Sequence Logo

Each element PWM(*i*,*j*) thus represents the log-likelihood ratio of observing character *i* at position *j* in a functional site compared with a random sequence. Figure 1 shows an example of a PFM, PPM and PWM and its corresponding sequence logo visualization [6].

Given a sequence of length *m*, the PWM score is obtained by summing over the PWM log-likelihood ratios at *m* positions, each time selecting the appropriate PWM element, i.e., the one that corresponds to the nucleotide in the sequence. Higher PWM scores express a better correspondence to the PWM model and thus a higher likelihood that the sequence represents a functional site.

In this work, we focus on the *PWM matching problem*: given an input sequence of length *n* (with typically *n*≫*m*), identify all *matches* or *occurrences* of a PWM, i.e. all subsequences for which the PWM score exceeds a user-defined threshold. These matches are then putative functional sites. The problem can be generalized to the case where the matches of multiple PWMs need to be identified, referred to as the *multiple PWM matching problem*. For long input sequences and/or a large number of PWMs, PWM matching is a compute-intensive problem that may require a large runtime. We briefly review the most important algorithmic approaches and refer to [7] for a more detailed overview.

A simple brute-force algorithm evaluates the PWM score at each possible starting position of the input sequence(s) and has a time complexity of *O*(*nm*). A simple improvement, called *lookahead scoring*, is to stop the computation of the PWM score as soon as it has been determined that the score threshold can no longer possibly be reached [8]. In *permuted lookahead scoring*, the PWM score is evaluated in such order that potential early termination is established as soon as possible. More advanced techniques for PWM matching usually involve generalizations of algorithms developed for exact pattern matching. They can be categorized as either online algorithms, that rely on the preprocessing of the PWM search matrices, or algorithms based on index structures, that rely on the preprocessing of the input sequence(s). In [9], PWM matches are identified using a depth-first traversal up to depth *m* of a suffix tree representation of the input sequence(s). This means that PWM score computations of repeated subsequences only need to be performed once and that computations can be partly reused between subsequences that share the same prefix. The use of lookahead scoring allows for the identification of subtrees that contain no PWM matches and that can be discarded from the search procedure. Similarly, the more space-efficient enhanced suffix array (ESA) [10] index structure is used in PoSSuMsearch [11, 12]. As index structures require *O*(*n*) memory, which can be costly in practice, much research has been devoted to online algorithms as well. In [13], index tables are constructed that contain precomputed (partial) PWM scores for all possible words or a short, fixed length. Additionally, potential similarity between multiple PWMs is exploited to accelerate the multiple PWM matching problem. In [14], the shift-add algorithm has been adopted for PWM matching. Similarly, in [15], the Morris-Pratt and Knuth-Morris-Pratt algorithms [16] are generalized to PWM matching. Finally, in [17], the Aho-Corasick, filtration and super-alphabet techniques developed for exact string matching are generalized to PWM matching, and further extended to the multiple PWM matching problem [18] and to higher-order PWM matching [19]. These algorithms are collectively implemented in the MOODS software package [20]. The use of graphics processing unit (GPU) architectures is investigated in [21]. The brute-force algorithm is parallelized over the different starting positions of the input sequence, as well as over the different PWMs, thus realizing important performance gains. Additionally, similarly to [13], index tables with precomputed partial PWM scores are used for further accelerate the search process.

These more complex PWM matching methods accelerate the (multiple) PWM matching problem by using various algorithmic techniques to avoid redundant computations and/or eliminate parts of the search space that are guaranteed not to contain PWM matches. The degree to which they can be successful largely depends on the PWM score threshold that is selected. When a high threshold is selected, the PWM matching problem resembles exact pattern matching for which efficient algorithms with *O*(*n*+*m*) time complexity exist. In contrast, for relaxed PWM score thresholds, the fraction of the search space that can be eliminated is small, and the time complexity eventually approaches the *O*(*nm*) complexity of the brute-force algorithm. As *m* takes a value between 5 and 15 for most practical applications, complex PWM matching algorithms may attain a speedup of approximately one order of magnitude over implementations of the brute-force algorithm.

In this work, we propose an alternative methodology to accelerate the PWM matching problem that is inspired by High Performance Computing techniques [22]. Rather than reducing the search space, we adopt the brute-force algorithm and reduce its runtime by expressing the PWM matching problem in terms of matrix-matrix products (MMP). MMPs can be evaluated with very high efficiency on modern CPUs for two reasons. First, they leverage SIMD (Single Instruction, Multiple Data) instructions that allow the same operation (multiplication, addition) to be performed on multiple data elements simultaneously. Second, MMPs can be implemented such that they maximally exploit spatial and temporal locality of reference, thus ensuring that most data accesses are satisfied from the CPU’s cache memory. As such, MMPs are among a select class of algorithms that can be evaluated with a performance that approaches the theoretical peak performance of the CPU. The latter is typically two orders of magnitude higher than what is effectively obtained by scalar (i.e., non-SIMD), compiler-generated code. High performance MMPs are provided through Basic Linear Algebra Subroutines (BLAS) [23] library implementations, for which most CPU vendors offer highly optimized implementations. Alternatively, open-source implementations such as ATLAS [24] or GotoBLAS [25] can be considered. The proposed method, named BLAMM (BLAS-Accelerated Motif Matching) inherits the advantages of the brute-force algorithm: it requires very little RAM, has a runtime that is independent of the selected PWM threshold(s) and is easy to implement and parallelize. We evaluated BLAMM on three recent generations of Intel CPU architectures and compared its performance with a naive implementation of the brute-force algorithm, MOODS (state-of-the-art online algorithm) and PoSSuMsearch (state-of-the-art index structure based algorithm) for different data sets and PWM threshold settings. Finally, we demonstrate BLAMM can easily leverage the computing power of graphics processing unit (GPU) architectures. To this end, we use the cuBLAS library [26] to efficiently perform the MMPs on the GPU rather than on the GPU. The performance of the GPU version of BLAMM is compared with TFM-CUDA, a state-of-the-art GPU implementation.

This paper is an extended version of the proceedings paper [27]. When comparing current benchmark results with the ones initially presented in [27], it should be understood that the performance of BLAMM in between both publications has improved significantly through various algorithmic and implementation improvements.

## Implementation

We consider the multiple PWM matching problem over a DNA alphabet. Starting from the brute-force algorithm, we express the evaluation of the PWM score at each possible starting position of the input sequence(s) in terms of matrix-matrix products (MMP). The procedure involves three matrices:

- A pattern matrix *P* that contains all of the PWMs.
- A sequence matrix *S* that encodes (part of) the DNA input sequence(s) using only elements 0 and 1.
- A result matrix *R* that is computed as *R*=sub(*S*)∗*P* and that contains the PWM scores of all PWMs at *some* positions in the input sequence(s). The routine sub(.) denotes that a submatrix of *S* is used.

**Fig. 2:**
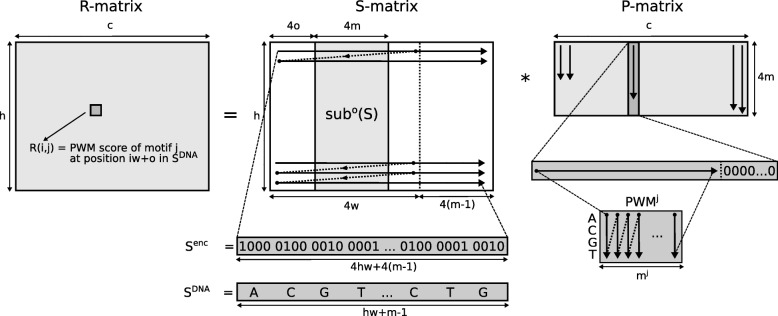
The result matrix *R* is computed as the matrix-matrix product of a submatrix of sequence matrix *S* and pattern matrix *P*. Each column of *P* represents a single PWM. Matrix *S* represents (part of) the input sequence. Each element in *R* contains a PWM score at some position in the input sequence

We now describe each matrix in detail. Figure 2 provides an overview of the algorithm.

### Pattern matrix *P*

The pattern matrix *P* is built once and remains constant during the execution of the algorithm. It has dimensions 4*m*×*c* where *c* denotes the total number of PWMs and *m*=max_*j*_(*m*^*j*^) refers to the maximum PWM length with *m*^*j*^ the length of PWM ^*j*^. Every column of *P* corresponds to a single PWM. The values in a column of *P* are obtained by unrolling the values of the corresponding PWM. For PWMs shorter than *m* characters, trailing zeros are appended to the corresponding column in *P*. Formally:

3 $$ P(i,j) = \left\{\begin{array}{ll} \text{PWM}^{j}(i~\text{mod}~4, \lfloor i / 4 \rfloor) & \quad 0 \le i < 4m^{j}\\ 0 & \quad i \ge 4m^{j} \end{array}\right.  $$

for all 0≤*j*<*c*.

In case PWM occurrences on both strands of the input sequence(s) need to be identified, *c* additional columns are added to matrix *P* that represent the reverse-complement of each PWM.

### Sequence matrix *S*

The sequence matrix *S* has dimensions *h*×4(*w*+*m*−1) where *h* and *w* can be arbitrarily chosen ≥1 and where *m* again represents the maximum PWM length. The matrix *S* is used to encode (part of) the input sequence(s) *S*^DNA^ of exactly *hw*+*m*−1 nucleotides. First, the string *S*^DNA^ is converted into an array *S*^enc^ of 4(*hw*+*m*−1) zeros and ones by simply replacing character A by 1000; C by 0100; G by 0010; and T by 0001. Formally:

4 $$ S^{\text{enc}}(i) = \left\{\begin{array}{ll} 1 & \quad S^{\text{DNA}}(\lfloor i/4 \rfloor) = A \wedge i~\text{mod}~4 = 0 \\ 1 & \quad S^{\text{DNA}}(\lfloor i/4 \rfloor) = C \wedge i~\text{mod}~4 = 1 \\ 1 & \quad S^{\text{DNA}}(\lfloor i/4 \rfloor) = G \wedge i~\text{mod}~4 = 2 \\ 1 & \quad S^{\text{DNA}}(\lfloor i/4 \rfloor) = T \wedge i~\text{mod}~4 = 3 \\ 0 & \quad \text{otherwise} \end{array}\right.  $$

for all 0≤*i*<4(*hw*+*m*−1). The matrix *S* is constructed from this temporary array as follows:

5 $$ S(i,j) = S^{\text{enc}}(4iw+j)  $$

for all 0≤*i*<*h* and 0≤*j*<4(*w*+*m*−1).

Every row in *S* contains a contiguous subarray of *S*^enc^ and thus encodes a substring of *S*^DNA^. The rightmost 4(*m*−1) elements of row *i* are identical to the leftmost 4(*m*−1) elements of row *i*+1. In other words, subsequent rows of *S* encode overlapping substrings of *S*^DNA^ with an overlap of *m*−1 characters.

### Result matrix *R*

The result matrix *R* has dimensions *h*×*c* and is computed as the matrix-matrix product of a submatrix of *S* and matrix *P*. Given an offset *o* with 0≤*o*<*w*, *R*^*o*^ is computed as follows:

6 $$ R^{o} = S(:,\,[4o,4(o+m)[) * P  $$

where the notation *S*(:, [4*o*,4(*o*+*m*)[) refers to the *h*×4*m* submatrix of *S* where the first column of the submatrix corresponds to the column with index 4*o* in *S*. Every element in *R*^*o*^ is thus computed as the dot product of (part of) a row in *S* and a column in *P*. The elements of *S* (zeros and ones) are multiplied with the elements of the PWM and thus generate the terms that, when added, correspond to the PWM score. As such, element *R*^*o*^(*i*,*j*) contains the score for PWM ^*j*^ at position *iw*+*o* in *S*^DNA^.


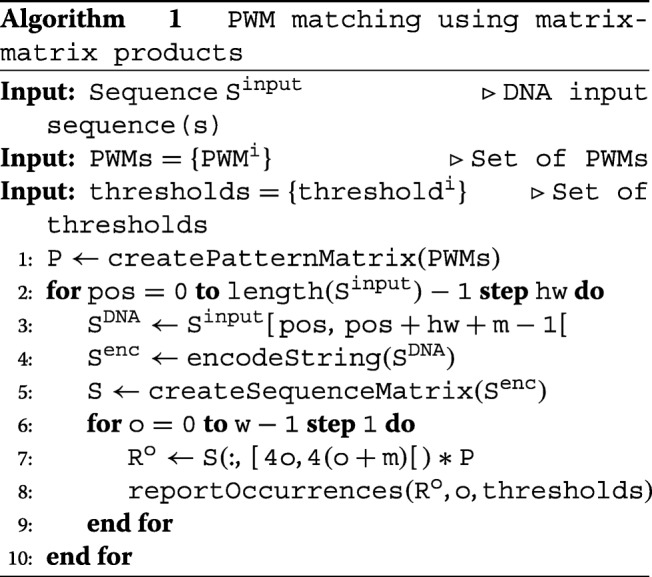


Algorithm 1 provides pseudocode for the entire workflow. In the outer for-loop, a portion of the input sequence(s) of length *hw*+*m*−1 is read into *S*^DNA^. In the inner for-loop, the PWM scores are exhaustively computed for all *c* PWMs at the *hw* first positions of *S*^DNA^. Therefore, the *S*^DNA^ strings at consecutive outer for-loop iterations overlap by *m*−1 nucleotides.

The time complexity of Algorithm 1 is given by:

7 $$\begin{array}{*{20}l}  T(n,m,c) &= \sum_{i=1}^{n/(hw)} \left[ O(hw) + \sum_{o=0}^{w-1}\left[ O(hmc) + O(n^{o,i}_{occ}) \right] \right] \end{array} $$

8 $$\begin{array}{*{20}l} &= \sum_{i=1}^{n/(hw)} \left[ O(hw) + O(hwmc) + O(n^{i}_{occ}) \right] \end{array} $$

9 $$\begin{array}{*{20}l} &= O(n) + O(nmc) + O(n_{occ}) \end{array} $$

where *n*_*occ*_ denotes the total number of motif occurrences identified. This further simplifies to *O*(*nmc*) which is the same time complexity as the brute-force algorithm. Note that the time complexity is indeed independent of the choice of parameters *h* and *w*.

The space complexity is given by $O(mc + h(w+m) + hc + n^{\text {buf}}_{occ})$, i.e. the space to store the three matrices and where $n^{\text {buf}}_{occ}$ denotes the maximum number of occurrences that are buffered in memory before they are spilled to disk (user-defined).

Note that in case the input data consists of multiple DNA sequences, these sequences can be concatenated when generating *S*^DNA^. With minimal extra bookkeeping, one can prevent the reporting of occurrences that span adjacent DNA sequences.

### Implementation details and performance considerations

The algorithm was implemented in C++ with support for multithreading using C++11 threads. The workload is easily split in independent subtasks by parallelizing the outer for-loop in Algorithm 1. Under the EREW-PRAM (Exclusive Read, Exclusive Write, Parallel Random-Access Machine) model, the time complexity using *p* parallel processes is given by:

10 $$\begin{array}{*{20}l} T_{p}(n,m,c) \le O(n) + O(nmc)/p + O(n_{occ})  \end{array} $$

where the *O*(*n*) term refers to data input and the *O*(*n*_*occ*_) term refers to data output. Concurrent disk access is indeed prohibited through the use of a mutual-exclusion (mutex) synchronization primitive. The ≤ sign in (10) reflects the fact that disk access and computations can overlap. As all datastructures involved are duplicated per thread, the memory complexity is given by $pO(mc + h(w+m) + hc + n^{\text {buf}}_{occ})$. This seems reasonable as the matrices require little memory in practice. The thread-local duplication of the read-only matrix *P* is done to avoid performance issues on non-uniform memory access (NUMA) architectures.

The BLAS sgemm routine [23] is used to evaluate the MMPs using single-precision computations. The runtime of BLAMM is largely governed by the time required to evaluate these MMPs and hence the quality of the BLAS library implementation.

Even though parameters *h* and *w* that govern the dimensions of matrix *S* can be arbitrarily chosen, for performance reasons, they should not be too small (as evaluating MMPs with small matrices is not very efficient) nor too large (to avoid excessive memory requirements). In our implementation, we set *h*=1000 and *w*=250 such that matrix *S* corresponds to a 1000×[1000+4(*m*−1)] matrix.

Note that BLAS routines have full support to specify submatrix ranges without any need to explicitly copy these submatrices onto separate data structures.

Finally, note that in [27], we described the algorithm in using the transpose matrices *R*, *P* and *S*. Even though both approaches are mathematically equivalent, we found that current representation has a higher performance in practice. This is likely due to the fact that, when using a column-major matrix layout (as is natural in BLAS), matrix sub ^*o*^(*S*) is represented as a linear array and strided-memory access is avoided.

### GPU version

Graphics processing units (GPUs) contain massively parallel processors able to perform certain tasks with higher efficiency than general-purpose CPUs. Using the cuBLAS [26] library, one can evaluate the MMPs on a GPU thus boosting the performance of BLAMM. To this end, the matrices *P* and *S* must be copied to the GPU memory. The constant pattern matrix *P* is copied only once, while a new sequence matrix *S* is copied during each outer for-loop iteration in Algorithm 1. To avoid having to copy the entire result matrix *R*^*o*^ from GPU memory to regular RAM after each MMP in the inner for-loop iteration, a kernel was developed in the CUDA programming language to identify the matrix indices (*i*,*j*) for which *R*^*o*^(*i*,*j*) exceeds the threshold score for PWM ^*j*^. This task, known as *stream compaction*, is also executed on the GPU itself. Only the set of indices (*i*,*j*) that correspond to actual PWM matches is copied from GPU to system RAM, thus minimizing the volume of the data that has to be transferred. The CPU itself is only responsible for preparing the *S* matrices and converting the indices (*i*,*j*) to formatted output that is written to disk. Again, by parallelizing over the outer for-loop in Algorithm 1, BLAMM supports the use of multiple GPU devices simultaneously.

The programming efforts required to enable GPU support are minimal as most tasks are handled by CUDA library calls (copying data between CPU and GPU, calling cublasSgemm,...). The only exception is the stream compaction kernel itself which consists of 7 lines of CUDA code.

### Partitioning of the *P*-matrix

The total number of floating point operations in BLAMM depends on the size of the input sequence(s) *n*, the total number of PWMs *c* and the length of the *longest* PWM *m*. Indeed, recall that PWMs with a length shorter than *m* are represented as a column in *P* by adding trailing zeros. In case many PWMs have a length that is substantially shorter than *m*, matrix *P* may contain a large fraction of zero elements. In turn, this may create significant overhead during the evaluation of the MMPs.

This overhead can easily be reduced by sorting the columns in *P* according to length of the PWM they represent and subsequently partitioning *P* into a number of tiles. The evaluation of *R*^*o*^=sub^*o*^(*S*)∗*P* can then be computed as a number of smaller MMPs as follows:.

11 $$ R^{o}(:,[\!c_{i},\,c_{i+1}[\!) \,=\, S(:,[\!4o,4(o+m_{i})[\!) * P([0,4m_{i}[,[c_{i},c_{i+1}[)   $$

where the interval [*c*_*i*_,*c*_*i*+1_[ corresponds to a subset of the columns in *R* and *P* and where *m*_*i*_ denotes the maximum PWM length in that range. When *m*_*i*_<*m* overhead is reduced.

**Fig. 3:**
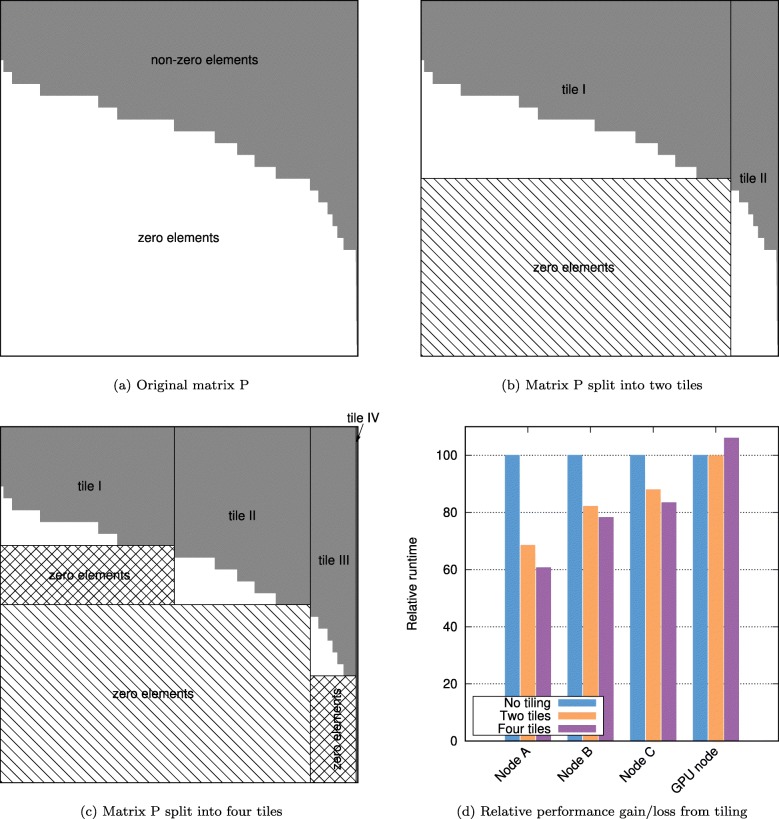
Example of a pattern matrix *P* containing 1404 JASPAR PWMs where many columns contain trailing zeros because of differences in length of the corresponding PWMs (panel **a**). Matrix *P* subdivided into two (panel **b**) and four (panel **c**) tiles. Relative performance gain/loss from tiling for different CPU and GPU architectures (panel **d**)

The idea is illustrated in Fig. 3 for the JASPAR dataset (see description below). The pattern matrix *P* represents 1404 PWMs with lengths between 5 and 30 and initially contains 62% zero elements (Fig. 3a). This means that more than half of the floating point operations executed during the MMP are redundant. Matrix *P* is divided into two tiles along some column index *c*_*i*_ (Fig. 3b). The leftmost tile then contains, at the bottom, a submatrix with only zero elements that can be discarded when evaluating the MMP. The column index *c*_*i*_ is selected such that the area of this zero submatrix is maximal. The process of matrix subdividing is applied recursively to the two tiles individually. The procedure is stopped when the area of the zero submatrix that can be discarded is smaller than a user-defined parameter. After two rounds of subdividing, four tiles are obtained (Fig. 3c) and 90% of the zero elements are discarded from *P*. Figure 3d shows the relative performance gains obtained by subdividing matrix *P*. Different CPU architectures (discussed further) benefit to varying degrees (40% to 18% reduction in runtime). The GPU architecture did not benefit from partitioning *P* (6% increase in runtime). The reason is that even though the total number of floating point operations is reduced, the process of tiling also yields smaller matrices for which the evaluation of the MMPs is less efficient. Current tiling algorithm appears to provide a good trade-off between reduction of zero fill while maintaining fairly large tiles. Note that this tiling algorithm is more efficient than the one we originally described in [27] where *P* was partitioned in slices with uniform width.

## Benchmark results

The performance of BLAMM was benchmarked against i) a naive implementation of the brute-force algorithm, ii) MOODS [20] as a state-of-the-art online algorithm, iii) PoSSuMsearch [11] as a state-of-the-art index structure based implementation and iv) TFM-CUDA [21], a state-of-the-art GPU-accelerated implementation. The naive implementation simply scans for PWM occurrences using three nested for-loops: one for-loop over the input sequence(s), a second for-loop over the different PWMs and a third for-loop to compute the PWM score. It has the same code quality standards as BLAMM to ensure a fair comparison.

A total of 1404 position frequency matrices with length between 5 and 30 were downloaded from the JASPAR CORE database [28]. The human genome reference genome (HG38) was retrieved from the GATK Resource Bundle [29]. Part of the benchmarks were run only on chromosome 1 (230 Mbp). For all tools, both strands of the DNA sequences were scanned for PWM occurrences. In the case of BLAMM, this is achieved by including the reverse complements of the PWM matrices as columns in matrix *P*. Thus effectively, 2808 PWM matrices were used in total.

Each tool computes the PWM score thresholds from a user-defined *p*-value. Smaller *p*-values give rise to higher thresholds and vice versa. Like MOODS, PoSSuMsearch and TFM-CUDA, BLAMM implements a dynamic programming algorithm to convert *p*-values to PWM score thresholds [30, 31]. Note that all tools use exact algorithms and should, in principle, produce identical results. Minor differences in output are due to i) slight differences in the process of converting *p*-values to PWM thresholds, ii) slight differences in the way pseudocounts are implemented, iii) differences in the way non-ACGT characters in the input sequences are handled. BLAMM’s CPU and GPU versions produce results identical to the naive brute-force implementation, regardless of the number of threads used. The exact command line arguments for each tool are listed in Additional file 1: Section S1.

The benchmarks were run on three generations of Intel CPU architectures, referred to as node A, B and C, respectively (see Table 1 for details). With each generation, the number of CPU cores increases (16, 24, 36, resp.) while the clock frequency shows a slight decrease (2.6, 2.5 and 2.3 GHz, resp.). Particularly relevant for BLAMM, however, are the SIMD capabilities of the different CPUs. Node A contains CPUs that support Advanced Vector Extensions (AVX) that operate on 256-bit AVX registers and that can thus contain 8 single precision (SP) floating point numbers. Each core is able to generate 16 single precision (SP) floating point operations (FLOPs) per CPU cycle: one 8-wide AVX addition and one 8-wide AVX multiplication. The CPUs in node B support AVX2 instructions which include fused multiply-add (FMA) operations that effectively perform a multiplication and addition in a single instruction. Hence, these CPU cores are able to generate 32 SP FLOPs per CPU cycle: two 8-wide FMA instructions. Finally, the CPU in node C has support for AVX-512 instructions which operate on 512-bit AVX registers that can hold 32 SP numbers again doubling the theoretical peak performance to 64 SP FLOPs per cycle. Table 1 also contains the specifications of a node with two nVidia 1080 Ti GPUs. In this case, as most of the workload is performed by the GPUs, the other system specifications (no. of CPU cores, SIMD capabilities) have only a marginal influence on the runtime.

**Table 1:** Configuration details of three generations of CPU nodes with AVX, AVX2 and AVX-512 SIMD support and one node with a dual GPU

	node A	node B	node C	GPU node
CPU	2 x Intel Xeon	2 x Intel Xeon	2 x Intel Xeon	2 x Intel Xeon
	E5-2670	E5-2680v3	Gold 6140	E5-2630v4
No. CPU cores	2 x 8 = 16	2 x 12 = 24	2 x 18 = 36	2 x 10 = 20
Clock freq.	2.6 GHz	2.5 GHz	2.3 GHz	2.2 GHz
Architecture	Sandy Bridge	Haswell-EP	Skylake	Broadwell
RAM	64 GB	64 GB	192 GB	128 GB
SIMD	AVX	AVX2	AVX-512	AVX2
GPU	-	-	-	2 x nVidia 1080 Ti

The C++ source code of BLAMM was compiled against the Intel Math Kernel Library (MKL) version 2018.1.163 which implements optimized BLAS routines for Intel CPUs. Depending on the CPU’s capabilities (AVX, AVX2, AVX-512), it automatically selects the most appropriate implementation. In all cases, multi-threading *within* the MKL was disabled. In other words, individual calls to sgemm were always executed in a single-threaded manner but multiple calls to sgemm are issued by different threads concurrently. The CUDA code was compiled with the nvcc compiler and linked against cuBLAS from the CUDA SDK version 8.0.

When performing the benchmarks with fewer threads than CPU cores, the remaining CPU cores were idle. Runtime (wall clock time) and peak resident memory use were measured using the Linux /usr/bin/time -v tool.

Table 2 shows the benchmark results of the brute-force, MOODS, PoSSuMsearch, TFM-CUDA and BLAMM algorithm on node C (36 CPU cores with AVX-512 support) when searching the occurrences of 1404 JASPAR PWMs on both strands of human chromosome 1 for two different *p*-values: 10^−5^ and 10^−4^. The brute-force algorithm, PoSSuMsearch (PWM matching module) and BLAMM support multithreading and were run using 1, 4, 16 and 36 CPU cores respectively. For those cases, the parallel speedup (acceleration factor w.r.t. the single-threaded run) and parallel efficiency (ratio of the parallel speedup and the no. of CPU cores used) are provided. PoSSuM requires an enhanced suffix array (ESA) to be constructed prior to the actual PWM matching; this runtime is reported separately and needs to be performed only once, independent of the *p*-value. The PoSSuM ESA construction step as well as MOODS do not have multithreading support. A more extensive version of this table is provided in Additional file 1: Table S3. Similar tables for node A and B are provided in Additional file 1: Tables S1 and S2, respectively.

**Table 2:**
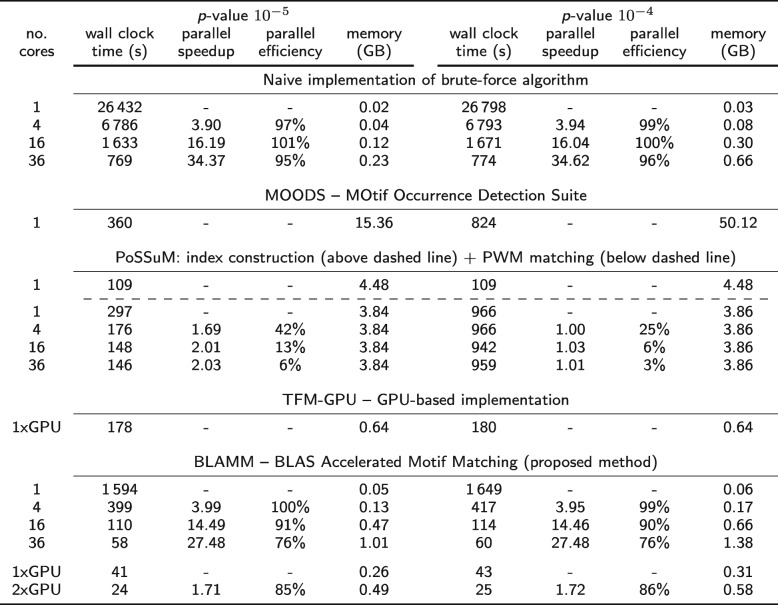
Benchmark results of the brute-force, MOODS, PoSSuMsearch and the proposed BLAMM algorithm on a 36-core Intel Skylake architecture (node C) and on a dual GPU system for TFM-CUDA and BLAMM (GPU mode)

In all cases, the occurrences of 1404 JASPAR PWMs were searched on both strands of human chromosome 1 for two different *p*-values (10^−5^ and 10^−4^)

Despite being the slowest, the naive implementation of the brute-force algorithm has only negligible memory use and shows nearly perfect scaling behavior. Additionally, its runtime is nearly independent of the *p*-value that is used. The minor differences can be attributed to the larger volume of output that has to be written to disk when more relaxed *p*-values are used. MOODS shows excellent runtime performance, taking into account that the software uses only a single CPU-core. However, its memory use is high, exceeding 50 GByte for a *p*-value of 10^−4^. Additionally, both the runtime and memory use depend on the selection of the *p*-value: more relaxed (i.e., higher) *p*-value settings result in additional resource requirements. The same is true for PoSSuMsearch: more relaxed *p*-value settings render it more difficult to eliminate parts of the search space that are guaranteed not to contain PWM occurrences. PoSSuMsearch has a runtime that is comparable to MOODS, albeit with a lower memory use. The software does not benefit much from multithreading with a parallel speedup of at most 2 despite the availability of 36 CPU cores. BLAMM shares the benefits of the brute-force algorithm: a low memory footprint, a runtime that is largely independent of the selected *p*-value, and very good multithreading scaling behavior with a parallel speedup exceeding 27 using 36 CPU cores. When comparing the single-core performance of BLAMM with MOODS and PoSSuMsearch, BLAMM is somewhat slower: it requires 2 to 4 CPU cores to achieve the same runtime. However, when allowed to make use of all available CPU cores, it is 6.2× to 13.7× faster than MOODS, while using only a fraction of the memory. When run on the dual GPU system, runtime is further reduced and PWM occurrences are identified in only 25s. Compared with TFM-CUDA, the GPU version of BLAMM is more than four times faster (single-GPU results). This again illustrates the high performance one can obtain when evaluating MMPs.

Table 3 shows the benchmark results when applying the algorithms to the entire human genome. Again, Additional file 1: Tables S4 and S5 contain the benchmark results on nodes A and B, respectively. The runtime of MOODS ranges from 36 min to over 3 h, depending on the *p*-value while the memory use ranges from 17.5 GB to 79 GB. Because of these high memory requirements, MOODS cannot be run on nodes A and B for this configuration. Due to the much larger size of the input sequence, memory requirements of PoSSuM also increase significantly. It takes over three hours to construct the ESA index structure and an additional 11 min to over three hours to identify the PWM matches. In contrast, BLAMM has a runtime that is nearly constant (13 min) and requires very little memory. On the GPU system, the runtimes of BLAMM are further reduced to under 5 min. Note that we were unable to run TFM-CUDA on this dataset, likely due to GPU memory restrictions.

**Table 3:**
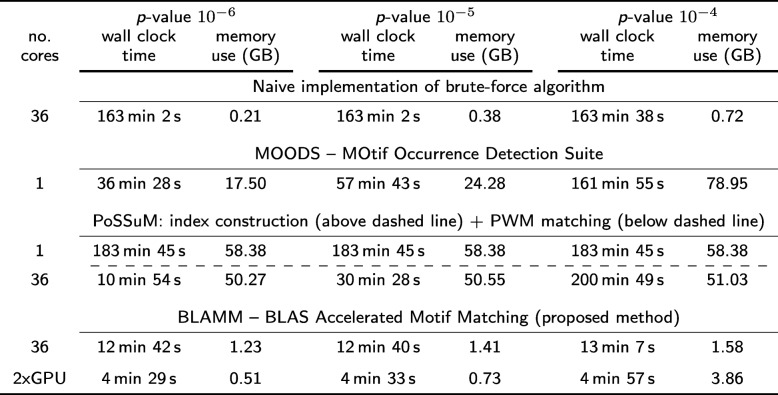
Benchmark results of the naive, MOODS, PoSSuMsearch and the proposed BLAMM algorithm on a 36-core Intel Skylake architecture (node C) and on a dual GPU system for BLAMM (GPU mode)

In all cases, the occurrences of 1404 JASPAR PWMs were searched on both strands of the entire human genome for three different *p*-values (10^−6^, 10^−5^ and 10^−4^)

**Fig. 4:**
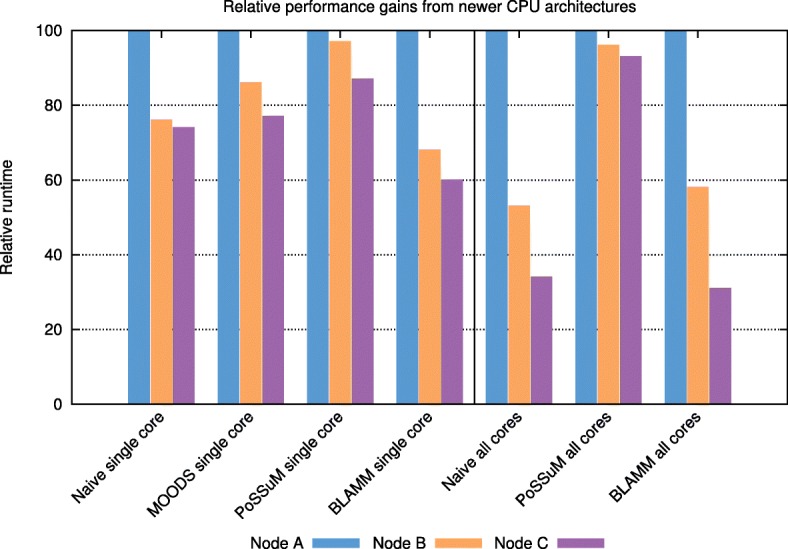
Relative performance gains from newer CPU architectures. For each tool, runtime was normalized to its runtime on node type A. Both single-threaded and multi-threaded results are shown. For the multi-threaded case, all available CPU cores were used on each node type (16, 24 and 36 for node type A, B and C, respectively)

Figure 4 shows the relative performance gains of the individual tools from newer CPU architectures. For each tool individually, we normalized its runtime (using chr. 1 and a *p*-value = 10^−5^) to the runtime obtained on the oldest CPU architecture (node A). Despite the decrease in CPU clock frequency, all tools benefit from newer architectures, albeit to varying degrees. Due to the introduction of AVX2 and AVX-512 in nodes B and C, respectively, BLAMM is able to benefit the most in terms of single-core performance. The availability of more powerful SIMD instructions in newer architectures automatically translates to faster MMPs and hence, lower runtimes. Also when considering the multithreaded case, BLAMM benefits the most. In that case, we observe that performance benefits not only from newer SIMD instructions, but also from an increasing number of CPU cores.

## Discussion

The PWM matching problem is a form of imprecise string matching. Under stringent settings (low *p*-value and hence, a high PWM score threshold), the problem approaches that of exact string matching for which efficient algorithms have been developed, both online and based on index structures. They reduce the complexity from *O*(*nm*) for a brute-force search to *O*(*n*+*m*). These ideas have been extended to PWM matching and implemented in tools such as MOODS and PoSSuMsearch. Nevertheless, for more relaxed *p*-values, these algorithms lose their ability to a-priori eliminate large parts of the search space and thus require more computational resources. In that respect, the proposed BLAMM algorithm has certain advantages:

- The runtime does not depend on the selected *p*-value. A small increase in runtime can be observed when relaxing the *p*-value, however, this is due to a higher number of PWM occurrences that have to be written to disk.
- The memory use of BLAMM is negligible and again independent of the selected *p*-value. Per CPU core, memory is required for the (relatively small) *S*, *P* and *R* matrices and to buffer occurrences before spilling them to disk. All data structures involved are thread-local and hence, BLAMM makes efficient use of multithreading up to a high number of CPU cores, even on non-uniform memory architectures (NUMA).
- The runtime of BLAMM is governed largely by the time required to evaluate the MMPs and hence, the quality of the BLAS implementation. Optimized BLAS libraries are provided by almost all CPU vendors. This enables BLAMM to make full use of current and future SIMD capabilities of CPUs, without needing to modify the source code of BLAMM itself. We have demonstrated this for AVX, AVX2 and AVX-512. As many applications in High Performance Computing and Artificial Intelligence benefit from improved BLAS performance, CPU vendors will surely continue along this path of developments in the future. For example, half-precision floating point computations are increasingly supported and might further improve BLAMM’s performance.
- The algorithm and its implementation are very simple.
- The algorithm is easily portable to GPUs and/or other co-processors or hardware accelerators. For GPUs, this achieved using the cuBLAS library to enable MMPs to be evaluated on massively parallel processors such as GPUs, thus offloading the CPU and boosting performance.

## Conclusions

We proposed and described BLAMM as a simple yet effective algorithm to identify position weight matrix occurrences in DNA sequences. The BLAMM algorithm is based on the brute-force algorithm in the sense that it exhaustively computes all PWM scores for all PWMs at each starting position of the input sequence(s). However, it does so with much higher efficiency by expressing all computations through matrix-matrix products (MMPs). It is well-known that MMPs are among a select class of algorithms that can be evaluated on modern, cache-based CPUs with a performance that approaches the theoretical peak performance of the CPU. Highly optimized MMP implementations are provided by CPU vendors through the BLAS library. BLAMM outperforms a naive implementation of the brute-force algorithm by a factor of 13 to 17. Additionally, it inherits the advantages of the brute-force algorithm, namely its simplicity, its negligible memory use, its ability to make very efficient use of multithreading and the fact that runtime and memory use are (largely) independent of the selected *p*-value or PWM score thresholds. Compared with state-of-the-art software package such as MOODS and PoSSuMsearch which implement more sophisticated search algorithms, BLAMM is a factor 2 to 4 slower when considering single-core performance. When using multithreading, BLAMM can be an order of magnitude faster than MOODS and PoSSuMsearch, while requiring only a fraction of their memory use. Finally, we presented an implementation of BLAMM on a GPU system that outperforms all CPU-based algorithms, as well as an existing GPU implementation by a large margin.

## Availability and requirements

**Project name:** BLAMM – BLAS-Accelerated Motif Matching **Project home page:**https://github.com/biointec/blamm**Operating system:** Platform independent **Programming language:** C++11 **Other requirements:** BLAS library, CUDA optional **License:** GNU GPL v3 **Any restrictions to use by non-academics:** none

## Supplementary information

**Additional file 1** BLAMM: BLAS-based Algorithm for Finding Position Weight Matrix Occurrences in DNA sequences on CPUs and GPUs.

## Data Availability

The motifs from the JASPAR database are publicly available [2]. The human genome reference genome (HG38) was retrieved from the GATK Resource Bundle [29].

## Abbreviations

ATLAS: Automatically tuned linear algebra software
AVX: Advanced vector extensions
BLAMM: BLAS-accelerated motif matching
BLAS: Basic linear algebra subroutines
CUDA: Compute unified device architecture
EREW-PRAM: Exclusive read exclusive write parallel random-access machine
FLOPs: Floating point operations
FMA: Fused multiply-add
GPU: Graphics processing unit
HPC: High performance computing
MKL: Math kernel library
MMP: Matrix-matrix product
MOODS: Motif occurrence detection suite
PFM: Position frequency matrix
PPM: Position probability matrix
PSSM: Position specific scoring matrix
PWM: Position weight matrix
SIMD: Single instruction multiple data
