# Supplementary material for: BLAMM: BLAS-based algorithm for finding position weight matrix occurrences in DNA sequences on CPUs and GPUs
Source: BMC Bioinformatics. 2020 Mar 11;21(Suppl 2):81. doi: 10.1186/s12859-020-3348-6 (PMC7068855; doi:10.1186/s12859-020-3348-6)
Supplement: Supplementary file 1 — Additional file 1 BLAMM: BLAS-based Algorithm for Finding Position Weight Matrix Occurrences in DNA sequences on CPUs and GPUs. [file 12859_2020_3348_MOESM1_ESM.pdf]

BLAMM: BLAS-based Algorithm for Finding Position Weight Matrix Occurrences in DNA sequences on CPUs and GPUs: Additional File 1

Jan Fostier

April 19, 2019

1 Command line arguments

- MOODS version 1.9.3

  Jaspar files were converted to raw PFM files. The exact background A,C,G,T probabilities were provided to MOODS.

```
python moods_dna.py --lo-bg <A C G T probabilities> --bg <A C G T probabilities>
    --batch --log-base 2 -p <p-value> -m *.pfm -s <sequence.fasta> > matches.txt
```

- PoSSuMsearch version 1.3

  Jaspar files were converted to Position Weight Matrices (PWM) in PoSSuM format. These PWMs were computed using the exact background A,C,G,T probabilities.

```
./mkvtree -dna -db <sequence.fasta> -indexname sequence.idx -tis -suf -lcp -skp -v
./possumfreqs -db sequence.idx > sequence.freq
./possumsearch-mt -j <numthreads> -pr motifs.possum -db sequence.idx -2 -esa
    -freq sequence.freq -lazy -pval <p-value> > matches.txt
```

- TFM-CUDA version 0.5

```
./pwmss -S -P <p-value exponent> -s <sequence.fasta> <dir-to-pwm>/*
```

- BLAMM version 1.0.0

```
./blamm dict sequences.mf
./blamm hist motifs.jaspar sequences.mf
./blamm scan -t <numthreads> -pt <p-value> -rc motifs.jaspar sequences.mf
```

  The GPU version can be enabled using the -c (‘CUDA’) flag.

```
./blamm scan -c -pt <p-value> -rc motifs.jaspar sequences.mf
```

- Naive implementation of brute-force algorithm

  The naive implementation can be enabled in BLAMM using the -s (‘simple mode’) flag.

```
./blamm scan -s -t <numthreads> -pt <p-value> -rc motifs.jaspar sequences.mf
```

2 Benchmark results

Tables 1, 2 and 3 show the benchmark results on node type A, B and C respectively (human chr. 1). Tables 4 and 5 show the benchmark results on node type A and B respectively (entire human genome). The table with the benchmark results on node C is incorporated in the main paper and is are therefore not reproduced here.

Table 1: Benchmark results of the naive, MOODS, PoSSuMsearch and the proposed BLAMM algorithm on a 16-core Intel Sandy Bridge architecture (node A). In all cases, the occurrences of 1404 JASPAR PWMs were searched on both strands of human chromosome 1 for three different p-values (10^{-6}, 10^{-5} and 10^{-4}).

	p-value 10^{-6}				p-value 10^{-5}				p-value 10^{-4}			
no. cores	wall clock time (s)	parallel speedup	parallel efficiency	memory (GByte)	wall clock time (s)	parallel speedup	parallel efficiency	memory (GByte)	wall clock time (s)	parallel speedup	parallel efficiency	memory (GByte)
	Naive implementation of brute-force algorithm											
1	35 479	-	-	0.02	35 560	-	-	0.02	35 691	-	-	0.03
2	17 789	1.99	100%	0.02	17 745	2.00	100%	0.03	17 797	2.01	100%	0.05
4	8 891	3.99	100%	0.03	8 914	3.99	100%	0.04	8 922	4.00	100%	0.08
8	4 466	7.94	99%	0.05	4 467	7.96	100%	0.07	4 476	7.97	100%	0.15
16	2 238	15.85	99%	0.10	2 236	15.90	99%	0.12	2 243	15.91	99%	0.28
	MOODS – MOtif Occurrence Detection Suite											
1	346	-	-	10.99	470	-	-	15.36	1 061	-	-	50.12
	PoSSuMsearch: ESA index construction (above dashed line) + motif search (below dashed line)											
1	121	-	-	4.48	121	-	-	4.48	121	-	-	4.48
1	148	-	-	3.83	346	-	-	3.84	1 143	-	-	3.86
2	89	1.66	83%	3.83	231	1.50	75%	3.84	1 099	1.04	52%	3.86
4	59	2.51	63%	3.83	175	1.98	49%	3.84	1 021	1.12	28%	3.86
8	47	3.51	39%	3.83	153	2.26	28%	3.84	1 002	1.14	14%	3.86
16	46	3.22	20%	3.83	154	2.25	14%	3.84	997	1.15	7%	3.86
	BLAMM – BLAS Accelerated Motif Matching (proposed method)											
1	2 661	-	-	0.04	2 674	-	-	0.04	2 758	-	-	0.05
2	1 331	2.00	100%	0.06	1 337	2.00	100%	0.07	1 378	2.00	100%	0.08
4	691	3.85	96%	0.10	694	3.85	96%	0.11	715	3.86	96%	0.14
8	353	7.54	94%	0.17	354	7.55	94%	0.19	364	7.58	95%	0.28
16	187	14.23	89%	0.34	186	14.38	90%	0.36	191	14.44	90%	0.54

Table 2: Benchmark results of the naive, MOODS, PoSSuMsearch and the proposed BLAMM algorithm on a 24-core Intel Haswell-EP architecture (node B). In all cases, the occurrences of 1404 JASPAR PWMs were searched on both strands of human chromosome 1 for three different p-values (10^{-6}, 10^{-5} and 10^{-4}).

	p-value 10^{-6}				p-value 10^{-5}				p-value 10^{-4}			
no. cores	wall clock time (s)	parallel speedup	parallel efficiency	memory (GByte)	wall clock time (s)	parallel speedup	parallel efficiency	memory (GByte)	wall clock time (s)	parallel speedup	parallel efficiency	memory (GByte)
	Naive implementation of brute-force algorithm											
1	27 128	-	-	0.02	27 172	-	-	0.02	27 272	-	-	0.03
2	13 565	2.00	100%	0.02	13 579	2.00	100%	0.03	13 611	2.00	100%	0.05
4	6 795	3.99	100%	0.03	6 807	3.99	100%	0.04	6 819	4.00	100%	0.09
8	3 408	7.96	100%	0.05	3 417	7.95	99%	0.07	3 424	7.96	100%	0.16
24	1 170	23.19	97%	0.13	1 174	23.14	96%	0.17	1 145	23.82	99%	0.44
	MOODS – MOtif Occurrence Detection Suite											
1	289	-	-	10.99	403	-	-	15.36	950	-	-	50.12
	PoSSuMsearch: ESA index construction (above dashed line) + motif search (below dashed line)											
1	110	-	-	4.48	110	-	-	4.48	110	-	-	4.48
1	148	-	-	3.83	341	-	-	3.84	1 312	-	-	3.86
2	91	1.63	81%	3.83	237	1.44	72%	3.84	1 079	1.22	61%	3.86
4	61	2.43	61%	3.83	175	1.95	49%	3.84	950	1.38	35%	3.86
8	54	2.74	34%	3.83	145	2.35	29%	3.84	903	1.45	18%	3.86
24	52	2.85	12%	3.83	154	2.21	9%	3.84	953	1.38	6%	3.86
	BLAMM – BLAS Accelerated Motif Matching (proposed method)											
1	1 820	-	-	0.04	1 829	-	-	0.05	1 890	-	-	0.06
2	911	2.00	100%	0.07	918	1.99	100%	0.08	949	1.99	100%	0.09
4	461	3.95	99%	0.12	463	3.95	99%	0.13	478	3.95	99%	0.17
8	248	7.34	92%	0.22	249	7.35	92%	0.25	256	7.38	92%	0.33
24	107	17.01	71%	0.64	107	17.09	71%	0.68	111	17.03	71%	0.97

Table 3: Benchmark results of the naive, MOODS, PoSSuMsearch and the proposed BLAMM algorithm on a 36-core Intel Skylake architecture (node C). In all cases, the occurrences of 1404 JASPAR PWMs were searched on both strands of human chromosome 1 for three different p-values (10^{-6}, 10^{-5} and 10^{-4}).

	p-value 10^{-6}				p-value 10^{-5}				p-value 10^{-4}			
no. cores	wall clock time (s)	parallel speedup	parallel efficiency	memory (GByte)	wall clock time (s)	parallel speedup	parallel efficiency	memory (GByte)	wall clock time (s)	parallel speedup	parallel efficiency	memory (GByte)
	Naive implementation of brute-force algorithm											
1	25 935	-	-	0.02	26 432	-	-	0.02	26 798	-	-	0.03
2	13 635	1.90	95%	0.02	13 281	1.99	100%	0.03	13 355	2.01	100%	0.05
4	6 726	3.86	96%	0.03	6 786	3.90	97%	0.04	6 793	3.94	99%	0.08
8	3 309	7.84	98%	0.05	3 329	7.94	99%	0.07	3 303	8.11	101%	0.15
16	1 655	15.67	98%	0.09	1 633	16.19	101%	0.12	1 671	16.04	100%	0.30
36	760	34.13	95%	0.18	769	34.37	95%	0.23	774	34.62	96%	0.66
	MOODS – MOtif Occurrence Detection Suite											
1	262	-	-	10.99	360	-	-	15.36	824	-	-	50.12
	PoSSuMsearch: ESA index construction (above dashed line) + motif search (below dashed line)											
1	109	-	-	4.48	109	-	-	4.48	109	-	-	4.48
1	125	-	-	3.83	297	-	-	3.84	966	-	-	3.86
2	85	1.47	74%	3.83	217	1.37	68%	3.84	1 056	0.91	46%	3.86
4	58	2.16	54%	3.83	176	1.69	42%	3.84	966	1.00	25%	3.86
8	49	2.55	32%	3.83	155	1.92	24%	3.84	930	1.04	13%	3.86
16	48	2.60	16%	3.83	148	2.01	13%	3.84	942	1.03	6%	3.86
36	47	2.66	7%	3.83	146	2.03	6%	3.84	959	1.01	3%	3.86
	BLAMM – BLAS Accelerated Motif Matching (proposed method)											
1	1 587	-	-	0.04	1 594	-	-	0.05	1 649	-	-	0.06
2	793	2.00	100%	0.07	798	2.00	100%	0.08	829	1.99	99%	0.10
4	398	3.99	100%	0.12	399	3.99	100%	0.13	417	3.95	99%	0.17
8	212	7.49	94%	0.23	213	7.48	94%	0.25	220	7.50	94%	0.34
16	111	14.30	89%	0.43	110	14.49	91%	0.47	114	14.46	90%	0.66
36	59	26.90	75%	0.96	58	27.48	76%	1.01	60	27.48	76%	1.38

Table 4: Benchmark results of the naive, MOODS, PoSSuMsearch and the proposed BLAMM algorithm on a 16-core Intel Sandy Bridge architecture (node A). In all cases, the occurrences of 1404 JASPAR PWMs were searched on both strands of the entire human genome for three different p-values (10^{-6}, 10^{-5} and 10^{-4}).

	p-value 10^{-6}		p-value 10^{-5}		p-value 10^{-4}	
no. cores	wall clock time	memory use (GB)	wall clock time	memory use (GB)	wall clock time	memory use (GB)
	Naive implementation of brute-force algorithm					
16	8 h 9 min 40 s	0.11	8 h 9 min 17 s	0.18	8 h 10 min 17 s	0.33
	MOODS – MOtif Occurrence Detection Suite					
1	46 min 21 s	17.50	1 h 12 min 33 s	24.28	DNC[1]	DNC[1]
	PoSSuMsearch: ESA index construction (above dashed line) + motif search (below dashed line)					
1	3 h 21 min 58 s	58.38	3 h 21 min 58 s	58.38	3 h 21 min 58 s	58.38
16	10 min 30 s	50.27	31 min 32 s	50.55	3 h 39 min 48 s	51.03
	BLAMM – BLAS Accelerated Motif Matching (proposed method)					
16	40 min 27 s	0.56	40 min 37 s	0.57	42 min 2 s	0.60

Table 5: Benchmark results of the naive, MOODS, PoSSuMsearch and the proposed BLAMM algorithm on a 24-core Intel Haswell-EP architecture (node B). In all cases, the occurrences of 1404 JASPAR PWMs were searched on both strands of the entire human genome for three different p-values (10^{-6}, 10^{-5} and 10^{-4}).

	p-value 10^{-6}		p-value 10^{-5}		p-value 10^{-4}	
no. cores	wall clock time	memory use (GB)	wall clock time	memory use (GB)	wall clock time	memory use (GB)
	Naive implementation of brute-force algorithm					
24	4 h 15 min 8 s	0.15	4 h 15 min 36 s	0.26	4 h 16 min 4 s	0.48
	MOODS – MOtif Occurrence Detection Suite					
1	41 min 23 s	17.50	1 h 6 min 13 s	24.28	DNC[1]	DNC[1]
	PoSSuMsearch: ESA index construction (above dashed line) + motif search (below dashed line)					
1	3 h 3 min 45 s	58.38	3 h 3 min 45 s	58.38	3 h 3 min 45 s	58.38
24	15 min 7 s	50.27	33 min 19 s	50.55	3 h 23 min 8 s	51.03
	BLAMM – BLAS Accelerated Motif Matching (proposed method)					
24	23 min 43 s	0.99	23 min 47 s	1.02	24 min 28 s	1.16

[0]DNC – Did Not Complete: run requires > 64 GB of RAM
